# Expression of G-Protein-Coupled Estrogen Receptor (*GPER*) in Whole Testicular Tissue and Laser-Capture Microdissected Testicular Compartments of Men with Normal and Aberrant Spermatogenesis

**DOI:** 10.3390/biology11030373

**Published:** 2022-02-26

**Authors:** Renata Walczak-Jędrzejowska, Ewa Forma, Elżbieta Oszukowska, Magdalena Bryś, Katarzyna Marchlewska, Krzysztof Kula, Jolanta Słowikowska-Hilczer

**Affiliations:** 1 Department of Andrology and Reproductive Endocrinology, Medical University of Lodz, Pomorska Str. 251, 92-213 Lodz, Poland; katarzyna.marchlewska@umed.lodz.pl (K.M.); krzysztof.kula@umed.lodz.pl (K.K.); jolanta.slowikowksa-hilczer@umed.lodz.pl (J.S.-H.); 2 Department of Cytobiochemistry, Faculty of Biology and Environmental Protection, University of Lodz, Pomorska Str. 141/143, 90-236 Lodz, Poland; ewa.forma@biol.uni.lodz.pl (E.F.); magdalena.brys@biol.uni.lodz.pl (M.B.); 3 II Clinic of Urology, Medical University of Lodz, Pabianicka Str. 62, 93-513 Lodz, Poland; joziorek@o2.pl

**Keywords:** estrogen receptor, G-protein-coupled estrogen receptor, obstructive azoospermia, non-obstructive azoospermia, Leydig cells, Sertoli cells, anti-Müllerian hormone, FSH, laser-capture microdissection

## Abstract

**Simple Summary:**

Nowadays, there is no doubt that estrogens play an important role in male reproduction, affecting testicular cell differentiation, proliferation, apoptosis and metabolism. It is also widely believed that intratesticular balance of androgens and estrogens is crucial for the testicular development and function and that the increased testicular estrogen production may be associated with spermatogenic failure. There is also growing epidemiological evidence that the exposure of men to endocrine disruptors demonstrating estrogenic activity (xenoestrogens) may lead to impairment of male fertility via interference with estrogen signaling pathways. Besides the two classical nuclear estrogen receptors, the membrane-bound G protein-coupled estrogen receptor (*GPER*) was described in human testicular tissue. However, there are little data on its expression in testes with disturbed spermatogenesis. In this study, we investigated the *GPER* expression pattern in biopsies of azoospermic men with complete and aberrant spermatogenesis. Our results showed an increased expression of the *GPER* in testes with impaired spermatogenesis. Moreover, they indicate a possible involvement of estrogen signaling through *GPER* in disturbed function of Sertoli cells—the cells that support spermatogenic process.

**Abstract:**

In this study, we retrospectively investigated *GPER* expression in biopsies of azoospermic men with complete (obstructive azoospermia—OA) and aberrant spermatogenesis (nonobstructive azoospermia—NOA). Each biopsy was histologically evaluated with morphometry. The testicular *GPER* expression was analyzed by the immunohistochemistry and RT-PCR technique in the whole testicular tissue and in seminiferous tubules and Leydig cells after laser-capture microdissection. In laser-microdissected compartments, we also analyzed transcriptional expression of selected Leydig (*CYP17A1*, HSD17B3, *StAR*) and Sertoli cell (*AMH*, *SCF*, *BMP4*) function markers. Immunohistochemical staining revealed expression of *GPER* in the cytoplasm of Leydig and Sertoli cells. Its stronger intensity was observed in Sertoli cells of NOA biopsies. The RT-PCR analysis of the *GPER* mRNA level unequivocally showed its increased expression in seminiferous tubules (i.e., Sertoli cells), not Leydig cells in NOA biopsies. This increased expression correlated positively with the transcriptional level of *AMH*—a marker of Sertoli cell immaturity, as well as FSH serum level in NOA but not in the OA group. Our results clearly demonstrate altered *GPER* expression in testes with primary spermatogenic impairment that might be related to Sertoli cell maturity/function.

## 1. Introduction

Normal male reproductive function is controlled by a complex endocrine regulation governed by the hypothalamus–pituitary–testes axis. Gonadotropins secreted by the pituitary control testicular function. Follicle stimulating hormone (FSH) controls the process of spermatogenesis, while luteinizing hormone (LH) controls the production of testosterone (T) by Leydig cells. Apart from FSH, T also, directly or by transformation to estradiol (E), supports spermatogenesis. The conversion of T to E is catalyzed by the enzyme aromatase. Classically, the localization and activity of aromatase in adult human testicular tissue had been reported in the cytoplasm of Leydig cells [1,2]. However, later studies also confirmed its expression in Sertoli, as well as germ cells [3,4].

Recent interest in the E function in testes is dictated by early observations that estradiol stimulates the number of spermatogonia [5], enhances the stimulatory effect of FSH on testicular maturation and contributes to precocious initiation of spermatogenesis [6]. These findings were further supported by other studies in which experimentally induced complete spermatogenesis was achieved by treating hypogonadal animals with E, thus extending the concept that E may have a direct or indirect stimulatory role in male gametogenesis [7,8]. In turn, E has been shown to stimulate germ cell survival in vitro in men [9]. Nowadays, there is no doubt that estrogens play an important role in male reproduction, affecting testicular cell differentiation, proliferation, apoptosis and metabolism [10]. It is also widely believed that intratesticular balance of androgens and estrogens is crucial for the testicular development and function in animals and humans and that the increased testicular E production may be associated with spermatogenic failure [10,11,12,13,14]. In agreement with the hypothesis of testicular dysgenetic syndrome (TDS), there is also growing epidemiological evidence that exposure of men to endocrine disruptors with estrogenic activity (xenoestrogens) during fetal, neonatal or adult life may impair male fertility via interference with the E signaling pathway [15,16,17].

Infertility is a problem that affects approximately 15% of couples, and the male factor is believed to contribute alone or in combination with female factor to about 50% of the cases [18]. Male infertility may result from anatomical or genetic abnormalities, endocrine disorders or systemic diseases, infections, trauma, iatrogenic injury or gonadotoxins [19,20], but the precise etiology of some of the cases remains unclear. One of the most severe conditions is the primary testicular failure, which results in non-obstructive azoospermia. In such cases, structural features, such as decreased seminiferous tubule diameters and both increased tubular membrane thickness and the number of Leydig cell in clusters forming micronodules, are frequently observed [21,22,23]. It is also well documented that apart from structural features, the deregulation of Sertoli and Leydig cell function, as evidenced by changes in hormones, proteins, enzymes, cell factors or receptors expression, was also reported in gonads of men with disturbed spermatogenesis [24,25,26,27,28,29].

There is also a large subset of studies revealing that men with primary spermatogenic impairment, besides compensatory increase in FSH secretion, may present decreased serum T levels, or normal T with slightly elevated LH, indicating a dysfunction of Leydig cells and normal-high level of E or elevated E/T ratio [22,30]. It was shown that the increased expression and/or activity of testicular aromatase may be responsible for increased intratesticular concentration of E accompanied by an augmented ratio of E/T in testes and serum of infertile men [4,24,30,31].

Estrogens exert their autocrine/paracrine action through two nuclear estrogen receptors (ERs): estrogen receptor 1 (ESR1) and estrogen receptor 2 (ESR2), which activate genomic signaling. It is now evident that the small pool of those receptors (approximately 5–10%) is also localized in the cell membrane and thus also mediates non-genomic signaling [10,32,33]. The ERs are widely distributed among different cells in both testicular compartments (seminiferous tubules and intertubular spaces) in different species, including human. Much data, often contradictory, have demonstrated their presence in somatic testicular cells (Leydig and/or Sertoli), as well as in germ cells [10,34,35,36,37,38]. In order to obtain conclusive results, the authors of one recently published study on cellular localization of ERs in human testes, used a combination of Western blotting, laser-capture-assisted cell picking, quantitative and qualitative RT-PCR and in situ hybridization, in addition to immunostaining. They concluded that ESR1 is present in germ cells and faintly in single Leydig cells, while ESR2 is found in germ cells and Sertoli cells [39]. In addition to ERs, a non-classical membrane-associated G-protein-coupled estrogen receptor (*GPER*), which mediates predominantly rapid non-genomic signaling of estrogens (mainly E), was discovered in several tissues, including testis [40]. Experimental studies on mice and rats have clearly confirmed that *GPER* signaling is involved in the regulation of germ and Sertoli cell proliferation and apoptosis [41,42,43], as well as Sertoli and Leydig cell function [44,45]. Additionally, *GPER* expression was also observed in a different region of rat’s epididymis, and its role in sperm maturation and storage was suggested [46]. In humans, there is a large subset of studies revealing a role of this receptor in pathogenesis and transformation of testicular tumors [47]. For example, it was shown that *GPER* is overexpressed in seminomas and may contribute to estrogen-dependent cancer cell proliferation [48]. However, there are little data on its role in the pathogenesis of male infertility. 

Until today, the analysis of *GPER* expression in men was limited mainly to testes with normal spermatogenesis [39,49,50]. In normal adult human testes, *GPER* was reported in the cytoplasm of somatic Sertoli and Leydig cells [39,49,50]. Some studies also reported its expression in spermatogenic cells (i.e., spermatogonia and spermatocytes) [49,50]. There are single data regarding *GPER* expression in human testicular tissues with impaired spermatogenesis. Bernardino et al. [51] reported that *GPER* mRNA abundance was higher in adult men with Klinefelter syndrome compared to control men, while the expression of ESR1 was unchanged and ESR2 was decreased. The authors suggested that *GPER* may play a role in the etiology of testicular abnormalities in this syndrome.

In this study, we address the question of whether the *GPER* expression pattern differs in biopsies with complete and aberrant spermatogenesis and how it is related to selected histological parameters and hormonal status of azoospermic men. To answer this question, an analysis of *GPER* mRNA expression was performed on the whole testicular tissue sample and the laser-microdissected testicular compartments, i.e., seminiferous tubule and Leydig cell clusters. As the primary testicular failure results in Sertoli and Leydig cells deregulation, we additionally analyzed gene transcripts of function markers of these cells in laser-microdissected compartments in relation to *GPER* mRNA expression. We selected the markers that might be potentially regulated by estrogen signaling. 

## 2. Materials and Methods

The study protocol was approved by the Bioethics Committee of the Medical University of Lodz (No. RNN/14/15/KE of 15 January, 2015). Due to its retrospective design and no anticipated effect on the prognosis or therapeutic management of the subjects whose tissue samples were included, informed consent was not obtained, and such a requirement was waived under the study protocol.

### 2.1. Characteristic of the Study Group

Testicular tissues embedded in paraffin blocks collected in the Department of Andrology and Reproductive Endocrinology of the Medical University of Lodz were used in this retrospective study. Testicular open biopsies were taken from 72 infertile men diagnosed with azoospermia during the period 2010–2014 as part of their medical diagnostic workup.

Data from medical histories concerning information on diseases that might influence fertility, testicular volume of the biopsied testis measured by ultrasound and serum hormone concentrations prior to testicular biopsy, were available. Hormonal measurements had been performed in one laboratory setting. Serum concentrations of FSH, LH, total testosterone (T) and total estradiol (E) were determined using enhanced chemiluminescence method for VITROS Immunodiagnostic System utilizing commercially available VITROS Reagent Packs for hormones, according to the manufacturer’s instruction (Ortho-Clinical Diagnostics Johnson and Johnson, New Brunswick, NJ, USA). Intra- and inter-assay coefficients of variations (CV) for measurements of FSH were <2.8% and <10.6%; for LH, they were <8.8% and <12.8%; for T, <3.8% and <7.4%; and for E, <6.6% and <8.8%, respectively. Additionally, T/LH and E/T ratios were calculated. 

The subjects were aged 23 to 41 years. They were not diagnosed with hypogonadotropic hypogonadism, chronic diseases, Klinefelter syndrome, microdeletions of AZF regions of Y chromosome or hormonally treated. 

### 2.2. Histological Analysis

All testicular biopsies were handled at the histological laboratory of the Department of Andrology and Reproductive Endocrinology, Medical University of Lodz. Testicular tissue samples were fixed in GR fixative (200 mL 37% formaldehyde, 40 mL acetic acid added to 1 L of 0.05 M, phosphate buffer, pH 7.4) [52], embedded in paraffin, and 5 μm thick serial sections were stained with hematoxylin and eosin. 

A histological analysis included a qualitative evaluation of seminiferous epithelium in 25 tubules of each testis with its subsequent quantitative analysis, according to the modified Johnsen score (JS) [53,54]. Next, the biopsies were classified into two groups: (1) obstructive azoospermia (OA)—biopsies with complete spermatogenesis (CS), which served as a control group (JS ≥ 8 points), and (2) non-obstructive azoospermia (NOA)—biopsies with a different pattern of spermatogenic failure, i.e., maturation arrest (ARR) and a mixed pattern (MIX) of disturbed spermatogenesis (JS < 8 points), as well as Sertoli cell-only syndrome (SCOS)—(JS ≤ 2 points) (Table 1). Additionally, biopsies included in the OA group were obtained from subjects with normal hormonal status (FSH 1–7 IU/mL, LH 1–8 mIU/mL, T 7–28 nmol/L, E up to 220 pmol/L).

A histological evaluation of biopsies, i.e., morphometric measurements of the tubule and semi-quantitative assessment of Leydig cells number, was performed with a light microscope (Eclipse E600; Nikon, Yokohama, Kawagawa, Japan) equipped with the image analysis system (NIS-Elements AR Ver. 3.2 Nikon, Japan). The slides were viewed and documented at 100×, 200× or 400× magnification (objective lens 10×) depending on the performed evaluation. 

The measurements of seminiferous tubules diameter (STD) and the thickness of tubular membrane (TM) were performed in at least 25 circular-shaped tubules. The following values were considered abnormal: <150 µm for STD and >10 µm for TM, based on data from previous studies [55,56].

A semi-quantitative assessment of the Leydig cells number was performed by a scoring system created by Guminska et al. [21,57] according to the number of Leydig cells observed in a particular intertubular space (IS):

- 0 Leydig cells—0 points
- 1–5 Leydig cells—1 point
- 6–10 Leydig cells—2 points
- 11–30 Leydig cells—3 points
- >30 Leydig cells (nodules of Leydig cells)—4 points

Leydig cells were recognized by their morphological features and location in the interstitial tissue. The morphological features include: (1) the eccentric, regular round or oval nucleus, usually with one or two prominent nucleoli; (2) the nucleus surrounded by abundant eosinophilic cytoplasm. The cells are usually located adjacent to the seminiferous tubules or near blood vessels (Supplementary Materials, Figure S1). In each biopsy specimen, all available triangular IS (no less than 50) were evaluated, and the mean number of points was calculated. A Leydig cells score (LC-score) > 2 points was considered abnormal.

### 2.3. Procedure of Tissue Sections Preparation for RNA Extraction

Extraction of mRNA was performed on whole testicular tissue sections from 72 biopsies. Additionally, we also performed mRNA extraction from the specific testicular compartments, i.e., seminiferous tubules and Leydig cell clusters. To achieve this, we used laser-capture microdissection (LCM) method with utilization of MMI CellCut/SmartCut system (Olympus/Molecular Machines & Industries, Eching, Germany). 

All procedures of tissue preparation were performed under RNase-free conditions by using RNaseZap (Thermo Fisher Scientific, Waltham, MA, USA) for equipment cleaning, and backed glassware and sterilized water treated with diethyl pyrocarbonate (DEPC) (Sigma Chemical Co., St. Louis, MO, USA) for specimen handling. Testicular tissues from paraffin blocks were cut into 10 µm thick sections adjacent to the sections used for histology and immunohistochemistry using a rotary microtome (Leica RM2155, Leica Microsystems, Deerfield, IL, USA) with a fresh disposable blade for each specimen.

For RNA extraction from the whole testicular tissue, after cutting, at least 4 sections were immediately carefully transferred into sterile RNase-free tubes (Thermo Fisher Scientific, Waltham, MA, USA) for further analysis (the day after). For RNA extraction from the laser-microdissected seminiferous tubules or Leydig cell clusters, the serial sections (at least 3–4) were carefully floated out on a warm DEPC-treated water bath (46 °C) and then mounted on RNase-free membrane slides (Molecular Machines &Industries, Glatsbrugg, Switzerland). To collect a similar tissue area of seminiferous tubules, as well as Leydig cell cluster (i.e., ~1 mm^2^), 4 to 8 membrane slides were prepared for each biopsy depending on the size of testicular section. Directly before the application of the LCM method, the membrane slides were incubated at 56 °C for 1 h, then deparaffinized and stained with hematoxylin and eosin using MMI SafeStain ampoules (H&E Staining Kit—PN 70302; Molecular Machines & Industries, Glatsbrugg, Switzerland) according to the manufacturer’s instructions. To add stabilization and secure the sections from contamination, a regular RNase-free microscopic slide was placed under the membrane slide. The seminiferous tubules or Leydig cell clusters from one membrane slide were cut within no longer than 1 h at room temperature under a 100× or 200× or 400× magnification (depending on the size of cut material) and collected to separate laser-microdissection tubes (500 µL) with adhesive lid (Molecular Machines & Industries Glatbrugg, Switzerland). Each LCM was preceded by optimization of laser power and spot distance to provide an effective dissection of marked areas. Usually, the laser setup was as follows: cut velocity or speed, between 10–15 µm/s; laser focus, 67–72; laser power, 72–90. The gaps in the tissue visually confirmed the success of the selected area capture after lid removal (Supplementary Materials, Figure S2). After microdissection, the collected seminiferous tubules or Leydig cell clusters were immediately dissolved in 150 μL buffer PKD from the RNeasy FFPE Kit (QIAGEN, Hilden, Germany) and stored at −80 °C until RNA extraction procedure.

### 2.4. RNA Extraction and RT-PCR

RNA extraction from the whole testicular tissue sections was performed using a RNeasy FFPE Kit (QIAGEN, Hilden, Germany) according to the manufacturer’s instructions. Before RNA isolation, the whole paraffin-embedded testicular sections were deparaffinized with xylene and then washed in ethanol to remove residual xylene from the sample. Deparaffinized blocks were immediately resuspended in 240 µL buffer PKD from the RNeasy FFPE Kit (QIAGEN, Hilden, Germany). To continue RNA extraction from LCM testicular compartments, tissue lysates from 4–5 caps of a single biopsy were pooled together. RNA concentration and purity were measured by absorbance through a BioPhotometer Plus UV/Vis Reader (Eppendorf, Hamburg, Germany). First-strand cDNAs were obtained by reverse transcription of 1 μg of total RNA, using a Maxima First Strand cDNA Synthesis Kit for RT-qPCR (ThermoFisher Scientific, Waltham, MA, USA) following the manufacturer’s protocol. 

Analysis of gene expression at the mRNA level was performed using TaqMan^®^ Gene Expression Assays (ThermoFisher Scientific, Waltham, MA, USA) according to the manufacturer’s instructions. The *RPS29* (ribosomal protein S29) gene was used as an internal control [58]. TaqMan^®^ Gene Expression Assays used in the study are presented in Table 2.

PCR reactions were performed in a 10 µL volume that included 5 µL of 2× TaqMan Universal PCR MasterMix (ThermoFisher Scientific, Waltham, MA, USA), 3.5 µL nuclease-free water (Sigma Aldrich, Munich, Germany), 1 µL cDNA template (50 ng) and 0.5 µL of the TaqMan^®^ Gene Expression Assay (ThermoFisher Scientific, Waltham, MA, USA). The RT-qPCR reaction was carried out using the Mastercycler ep realplex (Eppendorf, Hamburg, Germany). Relative RNA quantification was performed using the ΔCt method. ΔCt (Ct targeted gene—*RPS29*) values were recalculated into relative copy number values (number of target gene mRNA copies per 1000 copies of *RPS29* mRNA).

Possible cross-contamination of laser-microdissected Leydig cell cluster and somniferous tubules with adjacent cell types was also investigated. The transcriptional expressions of (1) FSH receptor (FSHR), which is exclusively expressed in Sertoli cells (assay code: Hs00174865_m1), (2) chymase (CMA1), specific to mast cells (assay code: Hs00156558_m1), (3) EGF-like module receptor 1 (EMR1), specific to macrophages (assay code: Hs00892591_m1) were quantified in laser-microdissected Leydig cell clusters [24]. The presence of 17α-hydroxylase/17,20-lyase (*CYP17A1*), specific to Leydig cells, was investigated in laser-microdissected seminiferous tubules [24]. In both compartments, the mRNA of α smooth muscle actin (ACTA2, assay code: Hs00426835_g1) was evaluated to analyze its cross-contamination with peritubular cells of the seminiferous tubular wall.

### 2.5. Immunohistochemical Expression of GPER

Immunohistochemical staining was performed on 5 µm thick sections. Prior to incubation with the primary antibody, testicular sections were deparaffinized and rehydrated using routine procedures, as described in our previous reports [55,57]. Antigen retrieval was accomplished by microwaving the sections in 10 nM citrate buffer (pH 6.0) at 750 W for 20 min. After cooling, to avoid non-specific staining, the sections were incubated with 3% hydrogen peroxidase for 10 min with successive incubation with blocking solution for 20 min (ImmPRESS peroxidase polymer detection kit, Vector Laboratories, Burlingame, CA, USA). Then, the slides were incubated overnight at 4 °C with primary antibodies against *GPER* (rabbit polyclonal, cat. no: orb85722, Biorbyt Ltd., Cambridge, UK; dilution: 1:100). The antibody was diluted with TBS. After incubation with the primary antibody, the reaction was further developed using commercially available visualization systems (ImmPRESS peroxidase polymer detection kit). Immunoreactivity was visualized by using diaminobenzidine chromogen (DAB) (Vector Laboratories, USA). Washing in TBS was performed between each step, except blocking solution and primary antibody. Finally, testis sections were counterstained with hematoxylin. In negative control sections, the primary antibody was omitted.

To quantify the intensity of immunohistochemical reaction in Leydig cells and in the tubules, we utilized the semi-quantitative method that was widely used in different types of tissues, including testes [59,60,61]. At least 10–15 Leydig cell clusters and 10–15 images of seminiferous tubules, in one representative biopsy slide [4], were captured under 40× objective lens, using a digital microscope camera (DS-Fi1C, Nikon), mounted on an optical microscope (Eclipse 600, Nikon, Japan) under the same light parameters and exposure time. Image processing and analyses were performed using the public domain ImageJ software (National Institute of Health, Bethesda, MD, USA, https://imagej.nih.gov/ij/download.html (accessed on 25 May 2021)). The intensity of *GPER* staining was expressed as a relative optical density (ROD) and was calculated using the formula described by Smolen [62]: ROD = ODspecimen/ODbackground = log (GLblank/GLspecimen)/log (log GLblank/GLbackground), where GL is the gray level for the stained area (specimen) and the unstained area (background), and blank is the gray level measured after removing the slide from the light path.

### 2.6. Statistical Analysis

The Statistica 13.1 software (StatSoft), licensed by the Medical University of Lodz, Poland, was used for statistical analysis. The Shapiro–Wilk test was applied to test the normality of obtained data. Due to a non-normal distribution of the majority of variables, non-parametric statistics were used. Medians and interquartile ranges (25th–75th percentiles) were used for descriptive data. The non-parametric Mann–Whitney U-test was used to evaluate the differences between groups. Spearman’s’s rank correlation coefficient (r) was used to evaluate correlations among continuous variables. A *p* value below 0.05 was considered statistically significant.

## 3. Results

### 3.1. Subjects and Histological Evaluation

**Figure 1:**
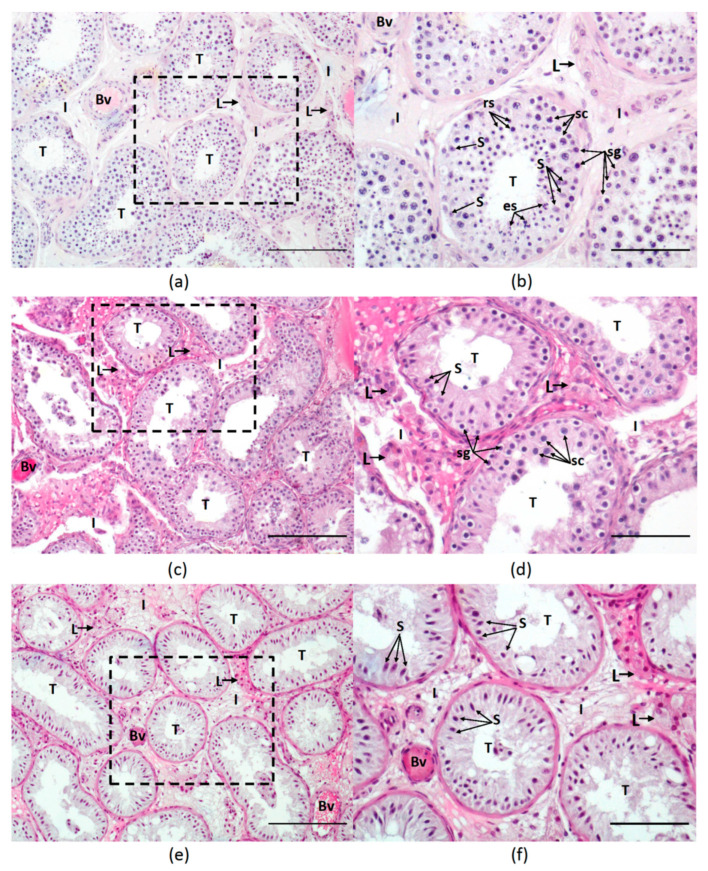
(**a**) Representative microphotograph of hematoxylin-eosin-stained histological sections of testicular biopsy from men with obstructive azoospermia. Seminiferous tubules (T) display a normal diameter and are lined with the seminiferous epithelium with complete spermatogenesis; between tubules, an interstitial space (I) is present with Leydig cell clusters (L) and blood vessels (Bv); (**b**) Higher magnification of the testicular area surrounded by the dash lines in (**a**); in seminiferous tubules, Sertoli cells characterized by large irregular nuclei with distinct nucleoli and extensive cytoplasmic processes extending from the basement membrane to the lumen of the tubule are present (S—Sertoli cell nucleus); Sertoli cell cytoplasm surrounds different types of germ cells: sg—spermatogonia, sc—spermatocytes, rs—round spermatids, es—elongated spermatids; (**c**) Representative microphotographs of the testicular biopsy from men with non-obstructive azoospermia presenting spermatogenic arrest at the spermatogonia and spermatocyte stage (spermatids are lacking), seminiferous tubules have a slightly decreased diameter, larger clusters of Leydig cells are present in the intertubular space; (**d**) Higher magnification of testicular area surrounded by the dash lines in (**c**) with marked testicular cells; (**e**) Representative microphotographs of the testicular biopsy from men with non-obstructive azoospermia presenting Sertoli cells-only syndrome; tubules have a decreased diameter, in the interstitial space, a larger cluster of Leydig cells is present, seminiferous tubules are devoid of germ cells and exclusively lined with Sertoli cells; notice the cytoplasm of Sertoli cells extends from the basement membrane to the lumen; (**f**) Higher magnification of testicular area surrounded by the dash lines in (**e**); (**a**,**c**,**e**)—magnification 200×, scale bar = 100 um; (**b**,**d**,**f**)—magnification 400×, scale bar = 50 µm.

Among 72 testicular biopsies, 19 presented complete spermatogenesis (CS), 25 presented an ARR/MIX pattern (n = 8 ARR and n = 17 MIX), and the remaining 28 presented tubules with SCOS. All the biopsies with disturbed spermatogenesis were included in one NOA group (n = 53). There were six ARR types at the spermatocyte and two at the round spermatid stage. Representative photomicrographs from biopsies with different histological patterns are presented in Figure 1. 

Among subjects with disturbed spermatogenesis, a history of cryptorchidism that was operated on during childhood was common (25%; 13/53), but in the remaining 40 cases, no serious andrological disturbances were noted. 

Data concerning clinical parameters and histological evaluation are presented in Table 3. There was no difference in age between subjects from the studied groups. In the group with impaired spermatogenesis (NOA), the testicular volume, as well as seminiferous tubule diameter (STD), were significantly lower than those in the OA group, while the thickness of tubular membrane (TM) was significantly higher. Abnormal STD (<150 µm) was present in 38% (20/53) of biopsies of NOA (56% of SCOS and 16% in ARR/MIX), while abnormal TM (>10 µm) was present in 28% (14/53) (40% of SCOS and 12% in ARR/MIX). With regard to the OA group, STD and TM values appeared to be normal in all biopsies.

The LC-score was significantly higher in NOA when compared to that of OA. The LC-score value above 2, which is considered abnormal, was observed in 23/53 (43%) in NOA biopsies (46% of SCOS and 44% of ARR/MIX). An abnormal LC-score was not observed in the OA group. 

### 3.2. Hormonal Profile

The serum hormonal profile of the subjects from the studied groups is shown in Table 4. Compared with OA, the majority of subjects with impaired spermatogenesis demonstrated significantly elevated FSH and LH levels. Although serum T concentrations were within the normal ranges in the NOA group and did not differ significantly from OA, the T/LH ratio was significantly decreased. The serum E level, as well as the E/T ratio, did not differ between the groups. To better characterize our subjects, we also investigated the relationship between the markers of spermatogenic damage (FSH and testicular volume) and Leydig cell function (T/LH ratio, LC-score). Considering all subjects, the T/LH ratio correlated negatively with the LC-score (r = −0.499, *p* < 0.001) and FSH level (r = −0.528, *p* < 0.001), and positively with the testicular volume (r = 0.323, *p* = 0.006). In addition, the LC-score correlated positively with FSH levels (r = 0.4293, *p* < 0.001) and negatively with the testicular volume (r = −0.487, *p* < 0.001).

### 3.3. Expression of GPER

#### 3.3.1. Expression of *GPER* mRNA in the Whole Testicular Tissue 

**Figure 2:**
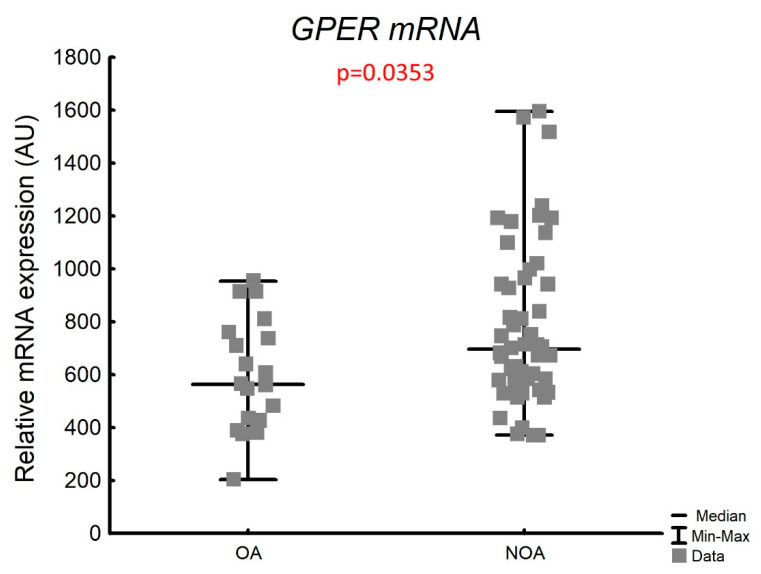
*GPER* transcriptional level in the whole testicular tissue in studied group. AU—arbitrary units (relative quantification normalized to the reference gene *RPS29* using the ΔCt method); OA—obstructive azoospermia with complete spermatogenesis; NOA—non-obstructive azoospermia with impaired spermatogenesis. *p* value—Mann–Whitney test.

The mRNA expression of *GPER* in the whole testicular tissue was detectable in all studied samples and significantly increased by 1.2-fold in the NOA group (median, range: 564.3; 203.1–953.9 in OA vs. 697.4; 372.2–1594.7 in NOA, *p* = 0.0353) (Figure 2).

When we analyzed a total group of subjects with impaired spermatogenesis (NOA), we observed a significant positive correlation between the *GPER* transcriptional expression and LC-score (r_s_ = 0.291 *p* = 0.0346), and FSH levels (r_s_ = 0.289 *p* = 0.0484). However, these correlations were not found in the control group. We also did not observe any significant correlation between the *GPER* transcriptional expression and the other hormone concentrations and histological parameters in both groups (Table 5).

#### 3.3.2. Expression of *GPER* mRNA in Laser-Microdissected Testicular Compartments

A seminiferous tubule laser microdissection (ST-LCM) was performed in 43 out of 72 biopsies: 27 from NOA (16-SCOS and 11-MIX/ARR) and 15 from OA (NOA-ST and OA-ST subgroups, respectively). A laser microdissection of Leydig cell clusters (LC-LCM) was performed in 27 out of 72 biopsies: 14 from NOA (7-SCOS and 7-MIX/ARR) and 13 from OA (NOA-LC and OA-LC subgroups, respectively). Data concerning clinical parameters, histological evaluation and serum hormonal levels of ST-LCM and LC-LCM subgroups are presented in Supplementary Materials (Tables S1–S4). We also performed a quantitative analysis of Sertoli cells number in biopsies subjected to seminiferous tubule laser microdissection. We did not observe differences between the number of Sertoli cells in OA-ST and NOA-ST (Supplementary Materials, Figure S3).

**Figure 3:**
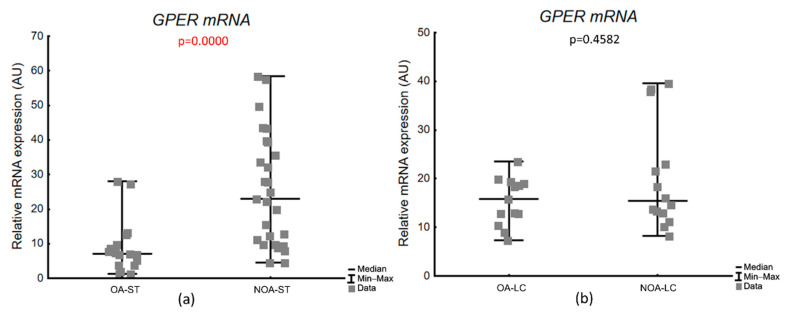
*GPER* transcriptional level in the laser-microdissected testicular compartments: (**a**) laser-microdissected seminiferous tubules (ST); (**b**) laser-microdissected Leydig cell clusters (LC). AU—arbitrary units (relative quantification normalized to the reference gene *RPS29* using the ΔCt method); *p* value—Mann–Whitney test.

mRNA expression of *GPER* in seminiferous tubule compartment and Leydig cell clusters was detectable in all studied samples from OA and NOA biopsies. We observed a significant, 3-fold increase in the studied transcript in seminiferous tubule compartment of the NOA-ST group in comparison with OA-ST (median, range: 7.5, 1.26–28.0 in OA-ST vs. 22.9, 4.6–58.4 in NOA-ST, *p* = 0.0000). There was no difference in *GPER* gene transcript expression between studied groups in the Leydig cells compartment (median, range: 15.8, 7.3–23.5 in OA-LC vs. 15.8, 8.3–39.6 in NOA-LC, *p* = 0.4582) (Figure 3).

Similarly to results obtained from the whole testicular tissue, *GPER* mRNA expression in laser-microdissected seminiferous tubules in NOA-ST correlated positively with FSH (r_s_ = 0.627, *p* = 0.0008), but additionally also with LH (r_s_ = 0.534, *p* = 0.0059) and negatively with the T/LH ratio (r_s_ = −0.435, *p* = 0.0296). We did not find significant correlations in OA-ST for the analyzed parameters (Table 6). Additionally, the *GPER* mRNA expression from laser-microdissected Leydig cell cluster did not correlate with clinical parameters, parameters of histological evaluation or hormone levels in the studied subgroups (Table 7).

An analysis of contamination with other cell types of our laser-microdissected compartments (i.e., seminiferous tubules and Leydig cell clusters) revealed marginal expression of mRNA corresponding to genes strongly and specifically expressed in cell types of seminiferous tubules and interstitium. The mean transcriptional expression of the Sertoli cell marker (FSHR) was very low in laser-microdissected Leydig cell clusters compared with the expression observed in laser-microdissected seminiferous tubules with complete spermatogenesis (Supplementary Materials, Figure S4a). The latter were barely contaminated with *CYP17A1* expression in comparison with its expression in laser-microdissected Leydig cell clusters (Supplementary Materials, Figure S5a). Additionally, laser-microdissected Leydig cell clusters demonstrated weak or no transcriptional expression of EMR1 (macrophage marker), CMA1 (mast cell marker) and ACTA2 (peritubular cell marker); their mean expression was comparable to that of FSHR (Supplementary Materials, Figure S4b). Similarly, the level of ACTA2 in laser-microdissected seminiferous tubules was marginal and comparable to the one observed for *CYP17A1* (Supplementary Materials, Figure S5b).

#### 3.3.3. Immunohistochemical Expression and Cellular Localization of *GPER*

**Figure 4:**
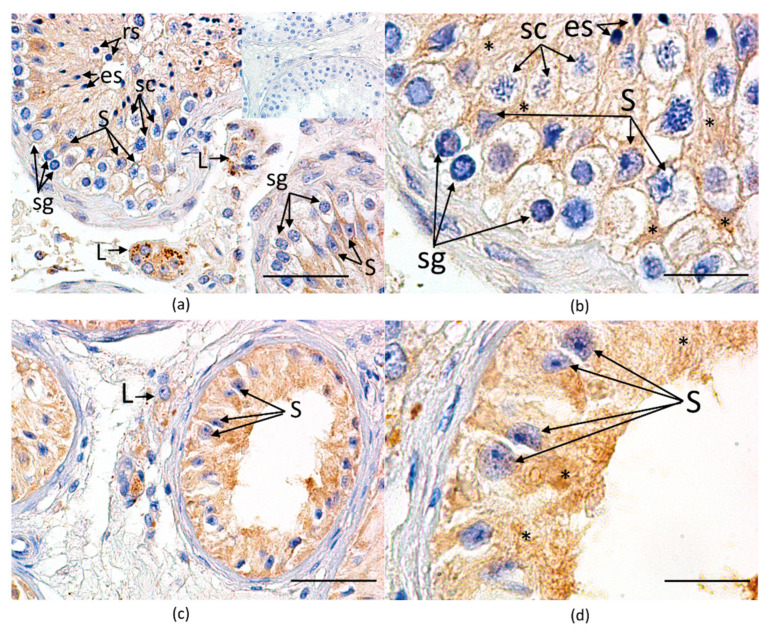
Representative microphotographs presenting immunohistochemical staining of *GPER* in histological specimens from (**a**) men with obstructive azoospermia presenting complete spermatogenesis (CS); and (**c**) from men with non-obstructive azoospermia presenting Sertoli cell-only syndrome; insert in (**a**)—negative control; (**b**,**d**)—higher magnifications of microphotographs (**a**,**c**), respectively. The color reaction of the studied antigen (*GPER*) is visible as a brown coloration of Leydig and Sertoli cell cytoplasm; L—Leydig cells cluster; S—Sertoli cell nuclei; sg—spermatogonia; sc—spermatocyte; rs—round spermatids; es—elongated spermatids; * — Sertoli cell cytoplasm in (**b**,**d**). Note the lack of color reaction in the rim of the cytoplasm surrounding nuclei of germ cells (**a**,**b**); (**a**,**c**)—magnification 400×, scale bar = 50 µm; (**b**,**d**)—magnification 1000×, scale bar = 20 um.

The staining *GPER* pattern was cytoplasmic and typical of seven-transmembrane receptors [63]. The color reaction was predominantly observed in the cytoplasm of Leydig and Sertoli cells (Figure 4). The used antibody against synthetic peptide derived between 261–365 amino acids of human *GPER* rendered the same staining pattern as another antibody, which recognizes epitope mapped against the third extracellular domain of the protein [49].

A semi-quantification of immunostaining intensity in Leydig cells and seminiferous tubules (i.e., Sertoli cells) was performed in a comparable number of clusters and tubules in each biopsy. The mean relative optical density of color reaction in Leydig cells did not differ between the groups and was increased in Sertoli cells in the NOA group (Table 8).

The mean ROD of color reaction in Leydig and Sertoli cells in LCM subgroups are presented in Table 9. Similarly to results obtained for the OA and NOA groups, we did not observe differences in intensity of immunostaining in Leydig cells between OA-LC and NOA-LC subgroups, while there was an increase in the ROD value in Sertoli cells from NOA-ST in comparison with OA-ST. 

We observed a significant positive correlation between the results of *GPER* expression at transcriptional and protein levels in both laser-microdissected testicular compartments. *GPER* mRNA expression in laser-microdissected seminiferous tubules correlated positively with ROD of Sertoli cells in both subgroups (r_s_ = 0.583, *p* = 0.0347 in OA-ST; r_s_ = 0.502, *p* = 0.0146 in NOA-ST). Similarly, *GPER* mRNA expression in laser-microdissected Leydig cell clusters correlated positively with ROD of Leydig cells in both subgroups (r_s_ = 0.577, *p* = 0.0494 in OA-LC; r_s_ = 0.614, *p* = 0.0254 in NOA-LC). 

### 3.4. Quantification of Sertoli and Leydig Cell Function Markers mRNA Transcripts in Laser-Microdissected Testicular Compartments 

#### 3.4.1. *AMH*, *BMP4*, *SCF* mRNA Transcripts in Laser-Microdissected Seminiferous Tubules 

**Figure 5:**
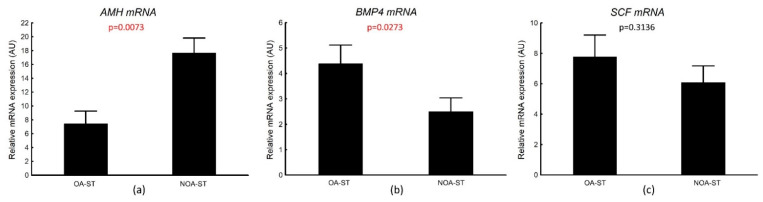
Transcriptional levels of: (**a**) anti-Müllerian hormone (*AMH*); (**b**) bone morphogenic protein 4 (*BMP4*); (**c**) stem cell factor (*SCF*) in laser-microdissected seminiferous tubules in the analyzed subgroups (OA-ST and NOA-ST). AU—arbitrary units (relative quantification normalized to the reference gene *RPS29* using the ΔCt method). Data are presented as mean ± SEM; *p* value—Mann–Whitney test.

mRNA expressions of Sertoli cell function markers in seminiferous tubule compartment were detectable in all studied samples from OA-ST and NOA-ST biopsies. The mRNA expression of *AMH* in laser-microdissected seminiferous tubules was significantly increased (mean ± SEM: 7.6 ± 2.0 in OA vs. 17.7 ± 2.1 in NOA, *p* = 0.0073), while *BMP4* was significantly decreased (mean ± SEM: 4.4 ± 0.7 in OA-ST vs. 2.5 ± 0.5 in NOA-ST, *p* = 0.0273) in the NOA-ST subgroup when compared with the OA-ST subgroup. We did not observe differences in *SCF* mRNA expression between the analyzed subgroups (mean ± SEM: 7.7 ± 1.4 in OA-ST vs. 6.0 ± 1.1 in NOA-ST, *p* = 0.3136) (Figure 5).

An analysis of correlations revealed a significant positive correlation between the *GPER* and *AMH* transcriptional expression in laser-microdissected seminiferous tubules in NOA-ST, but not in the OA-ST subgroup. No other correlations were observed between transcriptional expression of Sertoli cell markers and *GPER* in both groups (Table 10).

Similarly to *GPER* transcriptional expression, the *AMH* mRNA expression correlated positively with the FSH level in NOA-ST, but not in the OA-ST subgroup (r_s_ = 0.562, *p* = 0.0052 in NOA-ST; r_s_ = 0.166, *p* = 0.5872 in OA-ST). No significant correlation was observed between *AMH* transcripts and other hormone levels (data not shown).

#### 3.4.2. *StAR*, *CYP17A1*, *HSD173B* mRNA Transcripts in Laser-Microdissected Leydig Cells

**Figure 6:**
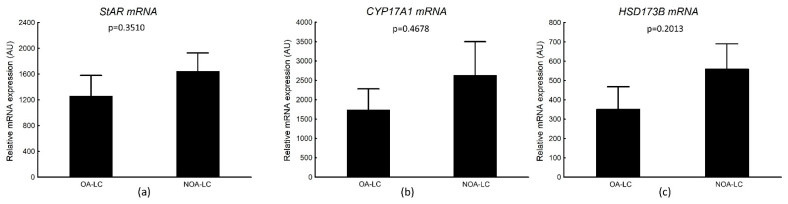
Transcriptional levels of: (**a**) steroidogenic acute regulatory protein (*StAR*); (**b**) 17α-hydroxylase/17,20-lyase (*CYP17A1*); (**c**) 17 β-hydroxysteroid dehydrogenase 3 (HSD17B3) in the laser-microdissected Leydig cell clusters in the analyzed subgroups (OA-LC and NOA-LC). AU—arbitrary units (relative quantification normalized to the reference gene *RPS29* using the ΔCt method). Data are presented as mean ± EM; *p* value—Mann–Whitney test.

The mRNA expressions of *StAR*, *CYP17A1*, *HSD173B* in Leydig cell clusters were detectable in all studied samples from OA and NOA biopsies. The mRNA expression of Leydig cell markers was not different in the analyzed subgroups (mean ± SEM: 1261.2 ± 319.3 in OA vs. 1645.0 ± 282.5 in NOA for *StAR*, *p* = 0.3510; 1862.6 ± 582.6 in OA vs. 2635.6 ± 864.6 in NOA for *CYP17A1*, *p* = 0.4678; 352.2 ± 115.6 in OA vs. 513.6 ± 132.9 in NOA for *HSD173B*, *p* = 0.2013) (Figure 6). We also did not observe any correlations between transcriptional expression of Leydig cell markers and *GPER* in both subgroups (Table 11). However, a correlation between *CYP17A1* and *GPER* mRNA was close to reaching statistical significance (*p* = 0.0529).

## 4. Discussion

To the best of our knowledge, this is the first study, which analyzed the *GPER* expression in a relatively large number of testicular biopsies of infertile men with primary spermatogenic failure. The strength of our study is the fact that the analysis of *GPER* mRNA expression was performed not only in the whole testicular tissue sample but also in laser-capture microdissected testicular compartments, i.e., separately in seminiferous tubules and Leydig cell clusters. It enabled us to reveal more accurately the alterations in *GPER* transcriptional expression in both studied compartments.

Available clinical data on the subjects from whom archival testicular biopsies were used for our study reveal that the majority of those with NOA clearly demonstrated testicular impairment, manifested by elevated FSH and LH concentrations [64,65]. A poor function of Leydig cells is accompanied by a decreased T/LH ratio, which in turn results from compensated elevation of LH [21,30,65]. No differences were observed in T and E levels. Although some earlier studies reported an elevation of E in the blood serum of infertile men [30,66], other studies did not confirm this observation [4,21,65]. This may be due to an extensive overlap in hormone levels between fertile and infertile men [30]. Additionally, the selection criteria (seminological vs. histological), as well as the type of control group (seminal normozoospermia vs. obstructive azoospermia with complete spermatogenesis), may also contribute to these differences [30,65,66]. Moreover, it was shown that men with primary spermatogenic impairment demonstrated an increased intratesticular E level, although no differences in its serum concentration could be detected [4].

Our results on *GPER* immunolocalization in somatic cells of testes with complete spermatogenesis (i.e., Sertoli and Leydig cells) correspond to those of a study by Rago et al. [49] and Fietz et al. [39]. However, some other studies also reported its presence in all germ cells [67] or diploid germ cells [50] or exclusively in peritubular cells [68]. The observed discrepancies may partly result from using different antibodies but may also be due to tissue fixation, an antigen retrieval method, as well as a detection method that may impact the accessibility of the antibody to the cell compartments, and subsequent visualization of the antigen as *GPER* was shown to reside in membranes of intracellular organelles, nucleus or plasma membranes [69,70]. 

Elevated mean *GPER* mRNA expression in the biopsies with disturbed spermatogenesis, obtained from men with NOA, is the main finding of this study. The application of the laser-capture microdissection technique and the analysis of the *GPER* transcriptional level in separate testicular compartments allowed us to unequivocally demonstrate altered overexpression of *GPER* mRNA in Sertoli, not in Leydig cells. Results obtained at the transcriptional level were also further confirmed at the protein level with utilization of immunohistochemical method. The semi-quantification of immunostaining intensity showed that the mean relative optical density of *GPER* protein was increased in Sertoli, not in Leydig cells, in NOA biopsies in comparison with the control group. Thus, we may assume that the elevation of the mean *GPER* mRNA expression in the whole testicular tissue sample from NOA biopsies results from both: (1) its increased expression in Sertoli cells, and (2) an increased size of Leydig cell clusters. This was further confirmed by an analysis of correlation between *GPER* expression and both hormone levels and features of histological evaluation. We observed positive correlations between *GPER* mRNA expression and LC-score in the whole testicular tissue sample, as well as FSH levels in the whole testicular tissue sample, as well as in laser-microdissected seminiferous tubule compartment. An increased size of Leydig cell clusters is a histological feature, commonly found in biopsies with impaired spermatogenesis in which a dysfunction of Leydig cells often occurs [23,65]. On the other hand, an elevated serum FSH level is a hormonal marker indicative of spermatogenic impairment and disturbed Sertoli cells function [71]. We cannot exclude *GPER* expression in other testicular cells using immunohistochemistry due to application of different primary antibodies and fixation methods. However, the close correlation between the intensity of immunohistochemical staining and *GPER* mRNA expression in testicular compartments suggests that the majority of *GPER* was localized in Sertoli, as well as in Leydig cells, without any post-transcriptional modification. To the best of our knowledge, only one study, apart from ours, analyzed *GPER* expression in testes with primary testicular failure. In accordance with our results obtained for the whole testicular sample, Brenardino et al. [51] have shown that *GPER* mRNA expression in testes of patients with Klinefelter syndrome was 12-fold higher in comparison with control men, which was confirmed at the protein level by a Western blot analysis. Unfortunately, the authors did not visualize the receptor in testicular tissue sections. However, a typical testicular histological picture of the majority of men with this genetic syndrome is well known to present tubules with Sertoli cells alone and extensive Leydig cell hyperplasia [72]. In our study, increased *GPER* mRNA expression was less pronounced than in patients with Klinefelter syndrome. However, this difference may be partly explained by the fact that endocrinological and histological abnormalities in our NOA patients might be milder compared to those observed in patients with Klinefelter syndrome [73,74]. What is interesting is the fact that overexpression of *GPER* receptors was also frequently reported in testicular tumors, and in seminomas, it was correlated with ERS2 downregulation. This inverse receptor expression pattern could reflect a switch in estrogen responsiveness [67,75]. 

A physiological role of *GPER* in the Sertoli cell function is well established in immature animals. It was suggested that *GPER* activation in these cells modulates the mechanism involved in the maintenance of their number [44,76]. However, we have no or little knowledge of its role in human Sertoli cells. In our study, we further investigated a relation between transcriptional expression of *GPER* and selected Sertoli cell markers in laser-microdissected seminiferous tubules. We analyzed the expression of *AMH*—one of the markers of Sertoli cell immaturity [28,77]—as well as *BMP4* and *SCF*, them being factors involved in germ cell differentiation, proliferation and survival [78]. *SCF* is a cytokine essential for spermatogenesis, and in testes, it is exclusively produced by Sertoli cells [79]. Animal studies showed that *SCF* is involved in gametogenesis by promoting mouse spermatogonia to differentiate into meiotic spermatocytes and round spermatids [80,81]. Moreover, its expression was increased under estrogen stimulation in rat seminiferous tubule cultured ex vivo [82]. *BMP4* is a growth factor that stimulates mouse stem cells to form progenitor spermatogonia and progenitors to express Kit—the receptor for *SCF*. *SCF* drives the final steps in differentiation of spermatogonia into type B spermatogonia [83,84]. In our study, we observed decreased expression of *BMP4* and no change in *SCF* mRNA level in seminiferous tubules with impaired spermatogenesis in comparison with those characterized with complete spermatogenesis, which corresponds to previous studies [85,86]. However, we did not find any correlation between transcriptional expression of *GPER* and *SCF* or *BMP4*.

The opposite results were observed for *AMH*, whose transcriptional level was increased in seminiferous tubule of NOA subgroup and correlated positively with *GPER* mRNA expression, as well as FSH serum levels. *AMH* is a hormone produced exclusively by Sertoli cells, and its main, well-recognized physiological role is the regression of Müllerian ducts in early fetal stages of males. Its production at high levels is sustained until puberty and then, along with Sertoli cell maturation, it declines during transition into adulthood. The low serum levels of *AMH* are characteristic for adult men. During the prepubertal period, *AMH* production is stimulated by FSH through the cAMP signaling pathway and the recruitment of transcriptional factors to *AMH* gene promoter [87]. In contrast, the physiological downregulation of *AMH* production results from androgen receptor activation and increased level of intratesticular androgen production in mature Sertoli cells [88,89]. Recently, a direct negative effect of androgens on the transcriptional activity of the *AMH* promoter has been shown in mice [90,91]. It is well documented that in a subset of testes with primary testicular failure with various histological patterns and etiology (including Klinefelter syndrome), an increased expression of *AMH* at testicular level is observed [28,77,92,93]. This altered expression of *AMH* in the adult seminiferous epithelium is regarded as a sign of either maintaining or regaining undifferentiated immature features of Sertoli cells [28,77]. Additionally, a few studies indicate that NOA men with impaired spermatogenesis demonstrate altered, diminished expression of androgen receptors in testes [25,29], Due to the retrospective design of our study, we could not obtain results of serum *AMH* concentration, which are not routinely conducted in diagnostic workup of men with infertility. However, one of the latest studies revealed that elevated serum *AMH* levels, in some men with NOA, are indicative of more severe primary testicular failure. Together with the *AMH*/T ratio, the *AMH* level served as an independent predictor of sperm retrieval from the testis [94]. Moreover, the very recent study has demonstrated that E upregulates *AMH* expression by increasing the activity of the h*AMH* promoter in the prepubertal Sertoli cell line SMAT1 (devoid of androgen receptor) and that this estrogen signaling might be mediated by ESR1, as well as *GPER* [95]. Expression of ESR1 in Sertoli cells of adult men with preserved and impaired spermatogenesis is controversial in comparison to *GPER* and ESR2 expression [39]. Thus, *GPER* together with ESR2 seem to be the main estrogen receptors in this cell type. It should be determined whether the observed changes in *GPER* expression in the present study, similarly to *AMH* expression, are a marker of the Sertoli cells maturational state or whether they are involved in the pathogenesis of observed disturbances. In this context, a possible mutual dependence between a disturbed function of Sertoli cells caused by their immaturity (increased *AMH* expression), hormonal imbalance (high FSH levels) and abnormal *GPER* expression in testes with spermatogenic impairment requires investigations in future research.

There is a number of studies reporting that *GPER* signaling might be involved in steroidogenesis regulation by E in animal and human Leydig cells [45,96]. It was shown that the activation of *GPER* lowers T levels in isolated Leydig cells of adult rat and human testicular tissue, with a statistically and clinically significant decrease in T production [45]. Another study also reported that aromatase overexpression in Leydig cells of some patients with primary gonadal failure, which elevates intratesticular E levels, may result in disturbed androgen biosynthesis, thereby accounting for Leydig cell dysfunction [24]. Another animal and in vitro study has demonstrated that *GPER* influences Leydig cell morphology and function in C57BL/6 mice and in MA-10 Leydig tumor cell line [96], while treatment of mice with *GPER* antagonist deregulated molecules controlling steroidogenesis (*StAR*, TSPO, PLIN) [97]. Recently, a link between adipokines and aromatase levels and/or signaling interactions has been observed in human Leydig cell tumors, which may suggest that increased aromatase expression may be a cause and consequence of Leydig cell tumor progression and/or initiation [98]. Thus, estrogen signaling through *GPER* might be considered a mechanism regulating E-dependent steroidogenesis in humans and animals. However, in the present study, we did not observe any differences in *GPER* mRNA transcripts expression in laser-microdissected Leydig cell clusters between the studied subgroups. The same observation was noted for mRNA expression of the analyzed Leydig cell markers, namely, *StAR* protein involved in cholesterol transport into inner mitochondrial membrane, and two steroidogenic enzymes: *CYP17A1* and HSD17B3. The enzymes are considered crucial for progression of the steroidogenic pathway. CYP17A catalyzes the conversion of pregnenolone to 17-OH pregnenolone and then to dehydroepiandrosterone (Δ5 pathway) or the conversion of progesterone to 17-OH progesterone and next to androstenedione (Δ4 pathway), while HSD17B3 catalyzes the conversion of androstenedione to testosterone [99]. Additionally, we did not observe any correlations between mRNA of *GPER* and Leydig cell function markers, although a correlation between *GPER* and *CYP17A1* transcripts expression was close to reaching statistical significance. Contrary to our results, Lardone et al. [27] showed that, in Leydig cell clusters obtained by laser-capture microdissection from men with SCOS and signs of Leydig cell dysfunction (i.e., T/LH ratio below 2.0), mRNA expression of *CYP17A1* was significantly increased in comparison to men with SCOS and without signs of Leydig cell dysfunction (i.e., T/LH above 2.0). Moreover, a positive correlation was observed between *CYP17A1* mRNA expression and intratesticular estrogen levels. Similarly, overexpression of HSD17B1 and *StAR* protein was observed in testes of men with Klinefelter syndrome, suggesting overactivation of the hormones’ biosynthetic pathway in Leydig cells [100]. The fact that our group of men was more heterogenous than the group selected in a study by Lardone et al. [27] and the group of men with Klinefelter syndrome might be a reason for discrepancies between those studies and our results [100]. 

## 5. Conclusions

This study is the first one to report increased expression of *GPER* in testicular tissue of men with primary testicular failure. An application of the laser-capture microdissection technique and the analysis of *GPER* transcriptional level in separate testicular compartments allowed us to unequivocally demonstrate altered overexpression of *GPER* mRNA in Sertoli, but not in Leydig cells, which was confirmed at the protein level. Furthermore, we also showed that this increased *GPER* expression in Sertoli cells was related to elevated FSH levels, as well as increased expression of *AMH* transcripts. Whether the observed change in *GPER* expression in the present study is a marker of Sertoli cells maturational state, or whether it is involved in the pathogenesis of observed disturbances, needs to be further elucidated. 

## Figures and Tables

**Table 1:** Histopathological description and Johnsen score (JS) of testicular biopsies.

Histological Group	Description	JS (Points) Median (Q1–Q3)
CS	All tubules with complete spermatogenesis or at least up to elongated spermatids	8.5 (8.1–8.8)
ARR/MIX	Tubules with germ cells until spermatogonia or spermatocytes or immature spermatids/single tubules with few elongated spermatids and others presenting maturational arrest at different stages or SCOS	4.5 (3.1–5.2)
SCOS	No tubules containing germ cells	2.0 (1.6–2.0)

ARR/MIX—maturation arrest or mixed pattern of disturbed spermatogenesis; CS—complete spermatogenesis; SCOS—Sertoli cell-only syndrome.

**Table 2:** TaqMan^®^ Gene Expression Assays used in the study.

Target Gene	Assay Code	Whole Tissue	ST-LCM	LC-LCM
*GPER*	Hs00173506_m1	+	+	+
*RPS29*	Hs03004310_g1	+	+	+
*AMH*	Hs01006984_g1	−	+	−
*BMP4*	Hs00370078_m1	−	+	−
*SCF*	Hs00931798_m1	−	+	−
*StAR*	Hs00986556_g1	−	−	
*CYP17A1*	Hs04981326_g1	−	−	+
*HSD173B*	Hs00609319_m1	−	−	+

*AMH*—anti-Müllerian hormone; *BMP4*—bone morphogenic protein 4; *CYP17A1*—17α-Hydroxylase/17,20-Lyase; *CYP19A1*—aromatase; *GPER*—G-protein-coupled estrogen receptor; *HSD173B*—17 β-hydroxysteroid dehydrogenase 3; LC-LCM—laser-microdissected Leydig cell cluster subgroups; *SCF*—stem cell factor; *StAR*—steroidogenic acute regulatory protein; ST-LCM—laser-microdissected seminiferous tubules subgroups; (+)—gene analysis performed; (−)—gene analysis not performed.

**Table 3:** Clinical parameters and results of histological evaluation in the studied groups.

	OA n = 19	NOA n = 53
Age (years)	32.0 (27.0–36.0)	31.0 (29.0–32.0)
Testicular volume (mL) *	15.2 (11.5–16.3)	10.1 (8.7–11.8) ^a^
STD (µm)	199.9 (188.5–208.0)	159.6 (139.5–181.8) ^a^
TM (µm)	6.5 (6.0–7.5)	8.0 (6.9–10.3)
LC-score (points)	1.5 (1.3–1.8)	2.2 (1.9–2.8) ^a^

Values are median (interquartile range); Mann–Whitney U test, ^a^ *p* < 0.05 with respect to OA; LC—Leydig cells; n—number of subjects; NOA—non-obstructive azoospermia with disturbed spermatogenesis; OA—obstructive azoospermia with complete spermatogenesis; STD—seminiferous tubules diameter; TM—thickness of tubular membrane; * volume of the biopsied testis.

**Table 4:** Serum hormonal profile in the studied groups.

	OA n = 19	NOA n = 53
FSH (mIU/mL)	3.5 (2.6–5.4)	13.5 (10.0–19.9) ^a^
LH (mIU/mL)	4.5 (3.4–5.4)	7.4 (5.8–9.8) ^a^
Testosterone (T) (nmol/L)	15.5 (10.9–20.1)	15.9 (11.9–21.1)
Estradiol (E) (pmol/L)	107.2(82.5–133.2)	110.1 (82.2–151.9)
T/LH ratio	4.0 (2.7–5.7)	2.4 (1.5–3.1) ^a^
E/T ratio	7.2 (4.8–9.2)	8.3 (6.9–10.2)

Values are median (interquartile range); Mann–Whitney U test, ^a^ *p* < 0.05 with respect to OA; n—number of subjects; NOA—non-obstructive azoospermia with disturbed spermatogenesis; OA—obstructive azoospermia with complete spermatogenesis.

**Table 5:** Spearman’s rank correlation (r_s_) between the *GPER* mRNA expression level and age, parameters of histological evaluation and hormonal levels in OA and NOA groups.

	*GPER* mRNA	
	OA n = 19 r_s_ (*p*)	NOA n = 53 r_s_ (*p*)
Age (years)	0.091 (0.7111)	0.106 (0.4486)
Testicular volume (mL) *	−0.142 (0.5620)	−0.283 (0.0586)
STD (µm)	0.331 (0.1653)	−0.106 (0.4525)
TM (µm)	0.058 (0.8105)	0.157 (0.2635)
LC-score	0.174 (0.4740)	0.291 (0.0346)
FSH (mIU/mL)	0.164 (0.5018)	0.289 (0.0484)
LH (mIU/mL)	−0.236 (0.3305)	0.055 (0.7001)
Testosterone (T) (nmol/L)	−0.254 (0.2928)	−0.175 (0.2089)
Estradiol (E) (pmol/L)	0.137 (0.6545)	0.183 (0.2915)
T/LH ratio	−0.061 (0.8054)	−0.110 (0.4391)
E/T ratio	0.157 (0.5599)	0.184 (0.2958)

LC—Leydig cells; n—number of subjects; NOA—non-obstructive azoospermia with disturbed spermatogenesis; OA—obstructive azoospermia with complete spermatogenesis; r—Spearman’s rank correlation coefficient; STD—seminiferous tubule diameter; TM—thickness of tubular membrane; * volume of the biopsied testis. Statistical significance in the Spearman’s rank correlation was reached when *p* < 0.05.

**Table 6:** Spearman’s rank correlation (r_s_) between the *GPER*-ST mRNA expression level and age, parameters of histological evaluation and hormonal levels in OA-ST and NOA-ST groups.

	*GPER* mRNA	
	OA-ST n = 16 r_s_ (*p*)	NOA-ST n = 27 r_s_ (*p*)
Age (years)	0.375 (0.1528)	−0.187 (0.3815)
Testicular volume (mL) *	−0.184 (0.4797)	−0.335 (0.1012)
STD (µm)	−0.208 (0.4238)	0.182 (0.3839)
TM (µm)	0.306 (0.2670)	0.291 (0.1582)
LC-score	0.294 (0.2513)	0.174 (0.4043)
FSH (mIU/mL)	−0.158 (0.6627)	0.627 (0.0008)
LH (mIU/mL)	0.164 (0.6504)	0.534 (0.0059)
Testosterone (T) (nmol/L)	0.170 (0.6382)	0.039 (0.8523)
Estradiol (E) (pmol/L)	0.371 (0.4684)	0.232 (0.3861)
T/LH ratio	−0.212 (0.5550)	−0.435 (0.0296)
E/T ratio	0.285 (0.4927)	−0.044 (0.8711)

LC—Leydig cells; n—number of subjects; NOA-ST—subgroup of men with non-obstructive azoospermia and disturbed spermatogenesis, whose biopsies were subjected to seminiferous tubule laser microdissection; OA-ST—subgroup of men with obstructive azoospermia and complete spermatogenesis, whose biopsies were subjected to seminiferous tubule laser microdissection; r—Spearman’s rank correlation coefficient; STD—seminiferous tubule diameter; TM—thickness of tubular membrane; * volume of the biopsied testis. Statistical significance in the Spearman’s rank correlation was reached when *p* < 0.05.

**Table 7:** Spearman’s rank correlation (r_s_) between the *GPER*-LC mRNA expression level and age, parameters of histological evaluation and hormonal levels in OA-LC and NOA-LC groups.

	*GPER* mRNA	
	OA-LC n = 13 r_s_ (*p*)	NOA-LC n = 14 r_s_ (*p*)
Age (years)	0.259 (0.4406)	−0.103 (0.7375)
Testicular volume (mL) *	0.082 (0.8084)	0.262 (0.4094)
STD (µm)	−0.301 (0.3701)	0.126 (0.6807)
TM (µm)	−0.329 (0.3528)	0.192 (0.5291)
LC-score	0.356 (0.2823)	0.162 (0.6156)
FSH (mIU/mL)	0.277 (0.4079)	−0.209 (0.4911)
LH (mIU/mL)	0.323 (0.3318)	−0.197 (0.5171)
Testosterone (T) (nmol/L)	0.059 (0.8626)	−0.461 (0.1123)
Estradiol (E) (pmol/L)	0.203 (0.6286)	0.103 (0.7769)
T/LH ratio	−0.123 (0.7186)	−0.192 (0.5291)
E/T ratio	0.460 (0.2125)	0.272 (0.4458)

LC—Leydig cells; n—number of subjects; NOA-LC—subgroup of men with non-obstructive azoospermia and disturbed spermatogenesis, whose biopsies were subjected to Leydig cell clusters laser microdissection; OA-LM—subgroup of men with obstructive azoospermia and complete spermatogenesis, whose biopsies were subjected to Leydig cell clusters laser microdissection; r—Spearman’s rank correlation coefficient; STD—seminiferous tubule diameter; TM—thickness of tubular membrane; * volume of the biopsied testis. Statistical significance in the Spearman’s rank correlation was reached when *p* < 0.05.

**Table 8:** Intensity of immunohistochemical staining for *GPER* expression as relative optical density (ROD) of DAB brown reaction product.

*GPER*	OA n = 19	NOA n = 53
ROD Leydig cells	10.4 (8.5–15.8)	10.3 (6.9–14.9)
ROD Sertoli cells	8.4 (5.9–9.8)	9.1 (8.7–10.3) ^a^

Values are median (interquartile range); Mann–Whitney U test, ^a^ *p* < 0.05 with respect to OA; LC—Leydig cells; n—number of subjects; NOA—non-obstructive azoospermia with disturbed spermatogenesis; OA—obstructive azoospermia with complete spermatogenesis; ROD—relative optical density.

**Table 9:** Intensity of immunohistochemical staining for *GPER* expression as relative optical density (ROD) of DAB brown reaction product in the biopsies subjected to laser microdissection.

***GPER* IHC**	**OA-LC** **n = 13**	**NOA-LC** **n = 14**
ROD Leydig cells	9.5 (5.7–14.1)	8.4 (7.0–14.9)
	**OA-ST** **n = 16**	**NOA-ST** **n = 27**
ROD Sertoli cells	8.2 (5.8–10.3)	10.9 (9.9–11.7) ^a^

Values are median (interquartile range); Mann–Whitney U test, ^a^ *p* < 0.05 with respect to OA-LC; LC—Leydig cells; n—number of subjects; NOA-LC—subgroup of men with non-obstructive azoospermia and disturbed spermatogenesis, whose biopsies were subjected to Leydig cell clusters laser microdissection; NOA-ST—subgroup of men with non-obstructive azoospermia and disturbed spermatogenesis, whose biopsies were subjected to seminiferous tubules laser microdissection; OA-LC—subgroup of men with obstructive azoospermia and complete spermatogenesis, whose biopsies were subjected to Leydig cell clusters laser microdissection; OA-ST—subgroup of men with obstructive azoospermia and complete spermatogenesis, whose biopsies were subjected to seminiferous tubules laser microdissection; ROD—relative optical density.

**Table 10:** Spearman’s rank correlation (r_s_) between the *GPER* and Sertoli cell markers mRNA expression level in OA-ST and NOA-ST subgroups.

	*GPER* mRNA	
	OA-ST n = 16 r_s_ (*p*)	NOA-ST n = 27 r_s_ (*p*)
*AMH*	0.167 (0.6914)	0.562 (0.0052)
*BMP4*	0.2029 (0.6997)	0.028 (0.8190)
*SCF*	−0.268 (0.4841)	0.4148 (0.0867)

*AMH*—anti-Müllerian hormone; *BMP4*—bone morphogenic protein 4; n—number of subjects; NOA-ST—subgroup of men with non-obstructive azoospermia and disturbed spermatogenesis, whose biopsies were subjected to seminiferous tubule laser microdissection; OA-ST—subgroup of men with obstructive azoospermia and complete spermatogenesis, whose biopsies were subjected to seminiferous tubule laser microdissection; *SCF*—stem cell factor. Statistical significance in the Spearman’s rank correlation was reached when *p* < 0.05.

**Table 11:** Spearman’s rank (r_s_) correlation between the *GPER* and Leydig cell markers mRNA expression levels in OA-LC and NOA-LC subgroups.

	*GPER* mRNA	
	OA-LC n = 13 r_s_ (*p*)	NOA-LC n = 14 r_s_ (*p*)
*StAR-LC*	0.320 (0.3369)	0.231 (0.5194)
*CYP17A1*-LC	0.379 (0.2491)	0.584 (0.0529)
*HSD17B3-LC*	0.407 (0.2762)	0.487 (0.1526)

*CYP17A1*—17α-hydroxylase/17,20-lyase; *GPER*—G-protein-coupled estrogen receptor; *HSD173B*—17 β-hydroxysteroid dehydrogenase 3; n—number of subjects; NOA-LC—subgroup of men with non-obstructive azoospermia and disturbed spermatogenesis, whose biopsies were subjected to Leydig cell cluster laser microdissection; OA-LC—subgroup of men with obstructive azoospermia and complete spermatogenesis, whose biopsies were subjected to Leydig cell cluster laser microdissection; *StAR*—steroidogenic acute regulatory protein. Statistical significance in the Spearman’s rank correlation was reached when *p* < 0.05.

## Data Availability

The data presented in this study are available upon request from the corresponding author.

## Supplementary Materials

The following supporting information can be downloaded at: https://www.mdpi.com/article/10.3390/biology11030373/s1, Figure S1: (a) Representative microphotograph of hematoxylin-eosin-stained histological sections of testicular biopsy from men with complete spermatogenesis from OA group; notice single Leydig cell (sL) near blood vessel (BV) and Leydig cell cluster (L-c, arrowheads) adjacent to seminiferous tubule (T); magnification 200×, scale bar = 50 µm (b) higher magnification of the testicular area surrounded by the dash lines in (a); Leydig cell cluster (L-c, arrowheads) adjacent to seminiferous tubule; notice the morphological features of Leydig cell: the eccentric, regular round or oval nucleus, usually with one or two prominent nucleoli and abundant eosinophilic cytoplasm (c); in the cytoplasm, the Reinke’s crystals are present (asterisk); (b)—magnification 400×, scale bar = 25 µm. Figure S2: Microphotographs of testicular tissue before and after laser-capture microdissection (LCM). (a) Seminiferous tubule (arrow) before and (b) after LCM; (c) Leydig cell clusters (arrowheads) before and (d) after LCM. Magnification (a,b)—200×; (c,d)—400×. Figure S3: (a) Number of Sertoli cells per seminiferous tubules in OA-ST and NOA-ST; data presented as mean± SEM; (b) microphotograph of testicular tissue with complete spermatogenesis; notice Sertoli cell nuclei (red asterisk) with extensive cytoplasm; magnification 600×, scale bar = 20 µm. Figure S4: Analysis of contamination with other cellular types in laser-microdissected Leydig cell clusters. (a) Transcriptional levels of follicle stimulating hormone receptor (FSHR) in microdissected seminiferous tubules with complete spermatogenesis (FSHR-ST) and Leydig cell clusters (FSHR-LC); (b) transcriptional levels of EGF-like module receptor 1 (EMR1), chymase (CMA1) and α smooth muscle actin (ACTA2) in laser-microdissected Leydig cell clusters (LC). AU—arbitrary units (relative quantification normalized to the reference gene *RPS29* using the ΔCt method). Data are presented as mean± SEM. Figure S5: Analysis of contamination with other cellular types in laser-microdissected seminiferous tubules. (a) Transcriptional levels of 17α-hydroxylase/17,20-lyase (*CYP17A1*) in laser-capture microdissected Leydig cell clusters (*CYP17A1*-LC) and seminiferous tubules with complete spermatogenesis (*CYP17A1*-ST); (b) transcriptional levels of α smooth muscle actin (ACTA2) in laser-microdissected seminiferous tubules (ST). AU—arbitrary units (relative quantification normalized to the reference gene *RPS29* using the ΔCt method). Data are presented as mean± SEM. Table S1: Clinical parameters and results of histological evaluation in the subgroups of men whose biopsies were subjected to seminiferous tubule (ST) laser microdissection. Table S2: Clinical parameters and results of histological evaluation in the subgroups of men whose biopsies were subjected to Leydig cell clusters (LC) laser microdissection. Table S3: Serum hormonal profile in the subgroups of men whose biopsies were subjected to seminiferous tubule (ST) laser microdissection technique. Table S4: Serum hormonal profile in the subgroups of men whose biopsies were subjected to Leydig cell cluster (LC) laser microdissection technique.
