# Supplementary material for: Expression of G-Protein-Coupled Estrogen Receptor (GPER) in Whole Testicular Tissue and Laser-Capture Microdissected Testicular Compartments of Men with Normal and Aberrant Spermatogenesis
Source: Biology (Basel). 2022 Feb 26;11(3):373. doi: 10.3390/biology11030373 (PMC8945034; doi:10.3390/biology11030373)
Supplement: Supplementary file 1 [file biology-11-00373-s001.zip › Figure S1.pdf]

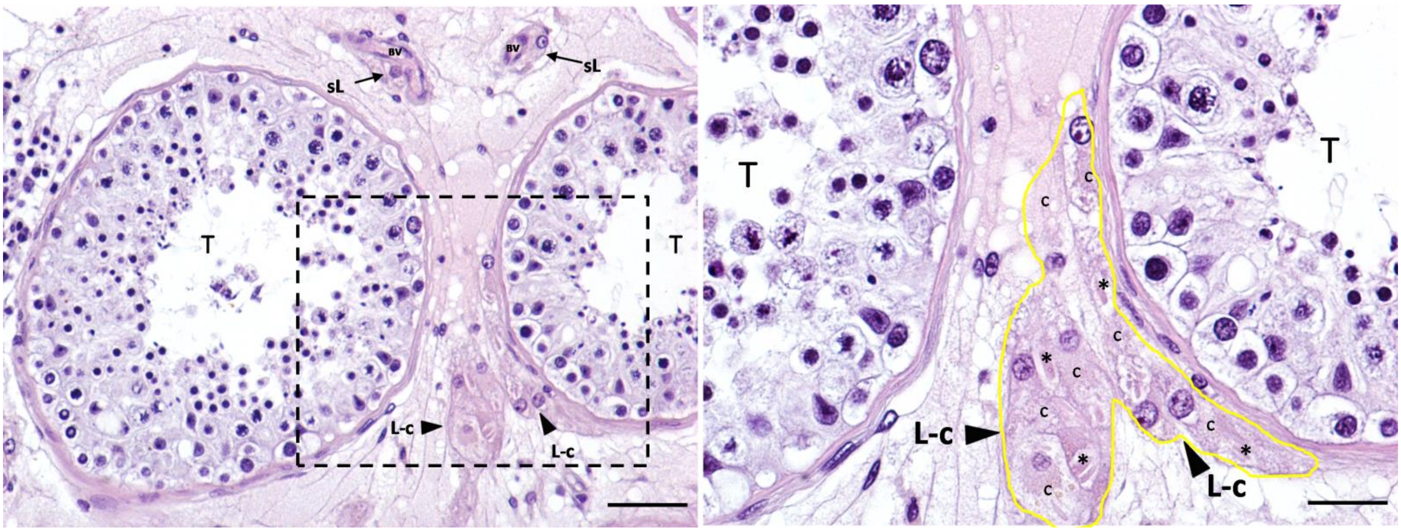

(a) (b)

Figure S1. (a) Representative microphotograph of hematoxylin-eosin-stained histological sections of testicular biopsy from men with complete spermatogenesis from OA group; notice single Leydig cell (sL) near blood vessel (BV) and Leydig cell cluster (L-c, arrowheads) adjacent to seminiferous tubule (T); magnification 200×, scale bar = 50 μm (b) higher magnification of the testicular area surrounded by the dash lines in (a); Leydig cell cluster (L-c, arrowheads) adjacent to seminiferous tubule; notice the morphological features of Leydig cell: the eccentric, regular round or oval nucleus, usually with one or two prominent nucleoli and abundant eosinophilic cytoplasm (c); in the cytoplasm, the Reinke's crystals are present (asterisk); (b)—magnification 400×, scale bar = 25 μm.
