# Supplementary material for: Expression of G-Protein-Coupled Estrogen Receptor (GPER) in Whole Testicular Tissue and Laser-Capture Microdissected Testicular Compartments of Men with Normal and Aberrant Spermatogenesis
Source: Biology (Basel). 2022 Feb 26;11(3):373. doi: 10.3390/biology11030373 (PMC8945034; doi:10.3390/biology11030373)
Supplement: Supplementary file 1 [file biology-11-00373-s001.zip › Figure S2.pdf]

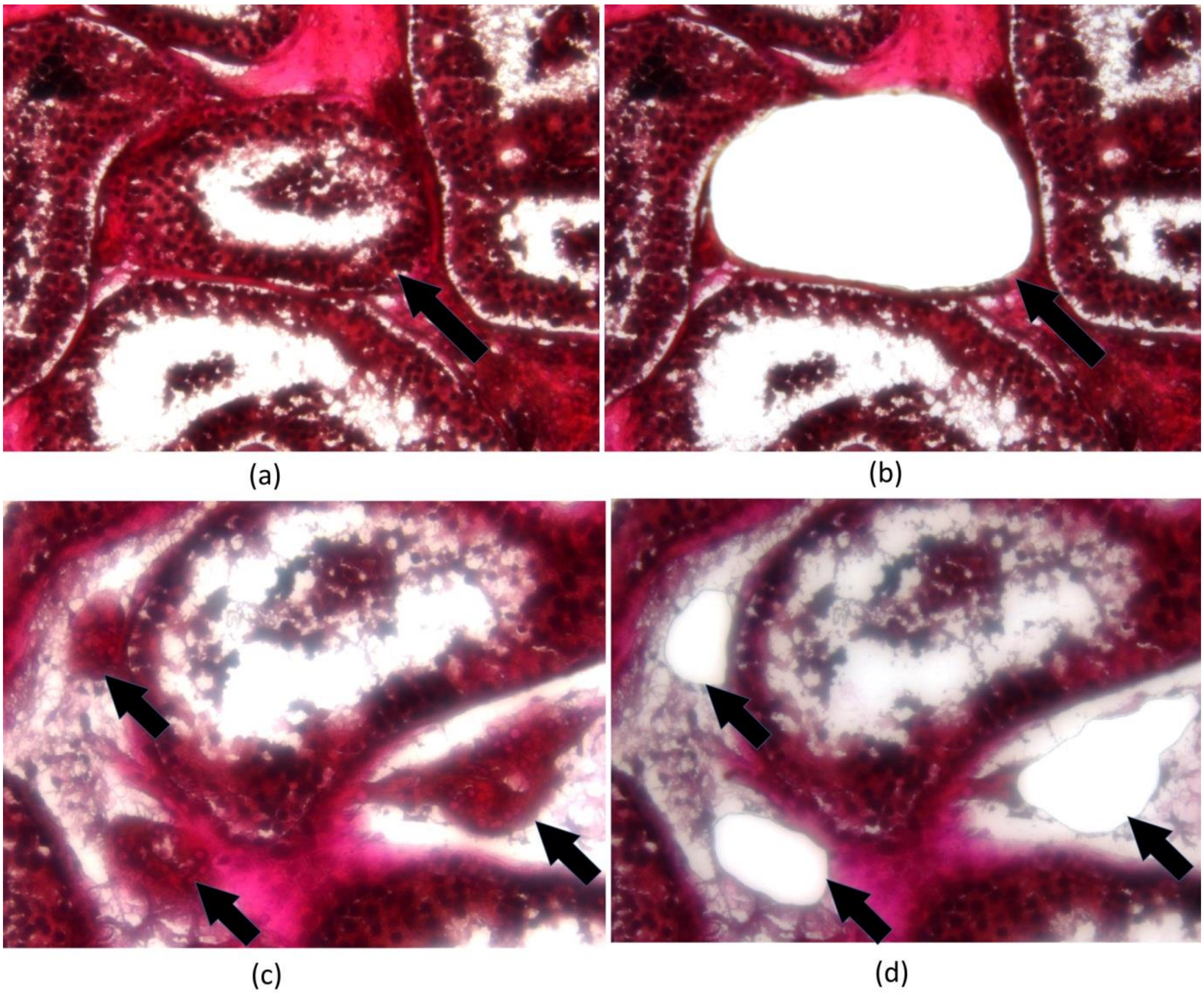

Figure S2. Microphotographs of testicular tissue before and after laser-capture microdissection (LCM). (a) Seminiferous tubule (arrow) before and (b) after LCM; (c) Leydig cell clusters (arrowheads) before and (d) after LCM. Magnification (a,b)—200×; (c,d)—400×.
