# Supplementary material for: Expression of G-Protein-Coupled Estrogen Receptor (GPER) in Whole Testicular Tissue and Laser-Capture Microdissected Testicular Compartments of Men with Normal and Aberrant Spermatogenesis
Source: Biology (Basel). 2022 Feb 26;11(3):373. doi: 10.3390/biology11030373 (PMC8945034; doi:10.3390/biology11030373)
Supplement: Supplementary file 1 [file biology-11-00373-s001.zip › Figure S3.pdf]

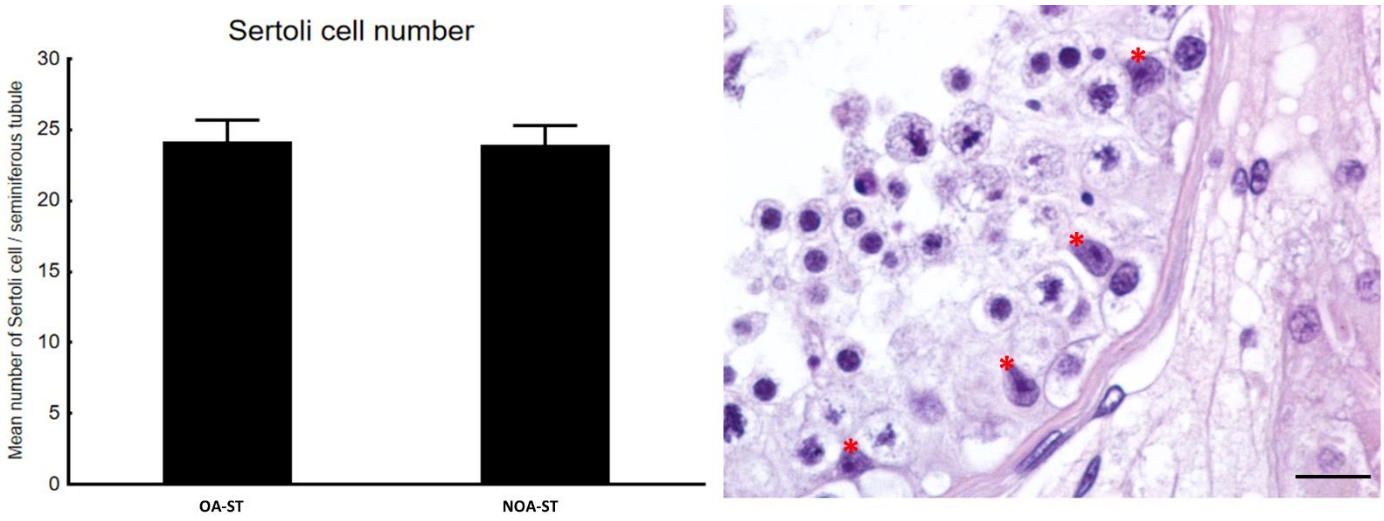

(a) (b)

Figure S3. (a) Number of Sertoli cells per seminiferous tubules in OA-ST and NOA-ST; data presented as mean± SEM; (b) microphotograph of testicular tissue with complete spermatogenesis; notice Sertoli cell nuclei (red asterisk) with extensive cytoplasm; magnification 600×, scale bar =20 μm.

Sertoli cell quantification

The number of Sertoli cells was counted from at least 10 round tubular cross-sections stained with hematoxylin-eosin from OA-ST (n=16) and NOA-ST (n=27) biopsies under a light microscope (Eclipse E600; Nikon, Kawagawa, Japan) equipped with the image analysis system (NIS-Elements AR Ver. 3.2 Nikon, Japan). The slides were viewed and documented at 600x magnification (objective 10x) The cells were identified for their typical characteristics: large irregular nuclei with distinct nucleoli and extensive cytoplasmic processes extending from the basement membrane to the lumen of the tubule (Figure S3B). Mean number of the cells per testicular tubule was calculated. The non-parametric Mann–Whitney U-test was used to evaluated differences between groups.

The mean number of Sertoli cells per seminiferous tubule did not differ between both groups (24.1±1.4 in OA-ST vs. 23.9±1.6 in NOA-ST) (Figure S3A).
