# Supplementary material for: Expression of G-Protein-Coupled Estrogen Receptor (GPER) in Whole Testicular Tissue and Laser-Capture Microdissected Testicular Compartments of Men with Normal and Aberrant Spermatogenesis
Source: Biology (Basel). 2022 Feb 26;11(3):373. doi: 10.3390/biology11030373 (PMC8945034; doi:10.3390/biology11030373)
Supplement: Supplementary file 1 [file biology-11-00373-s001.zip › Figure S4.pdf]

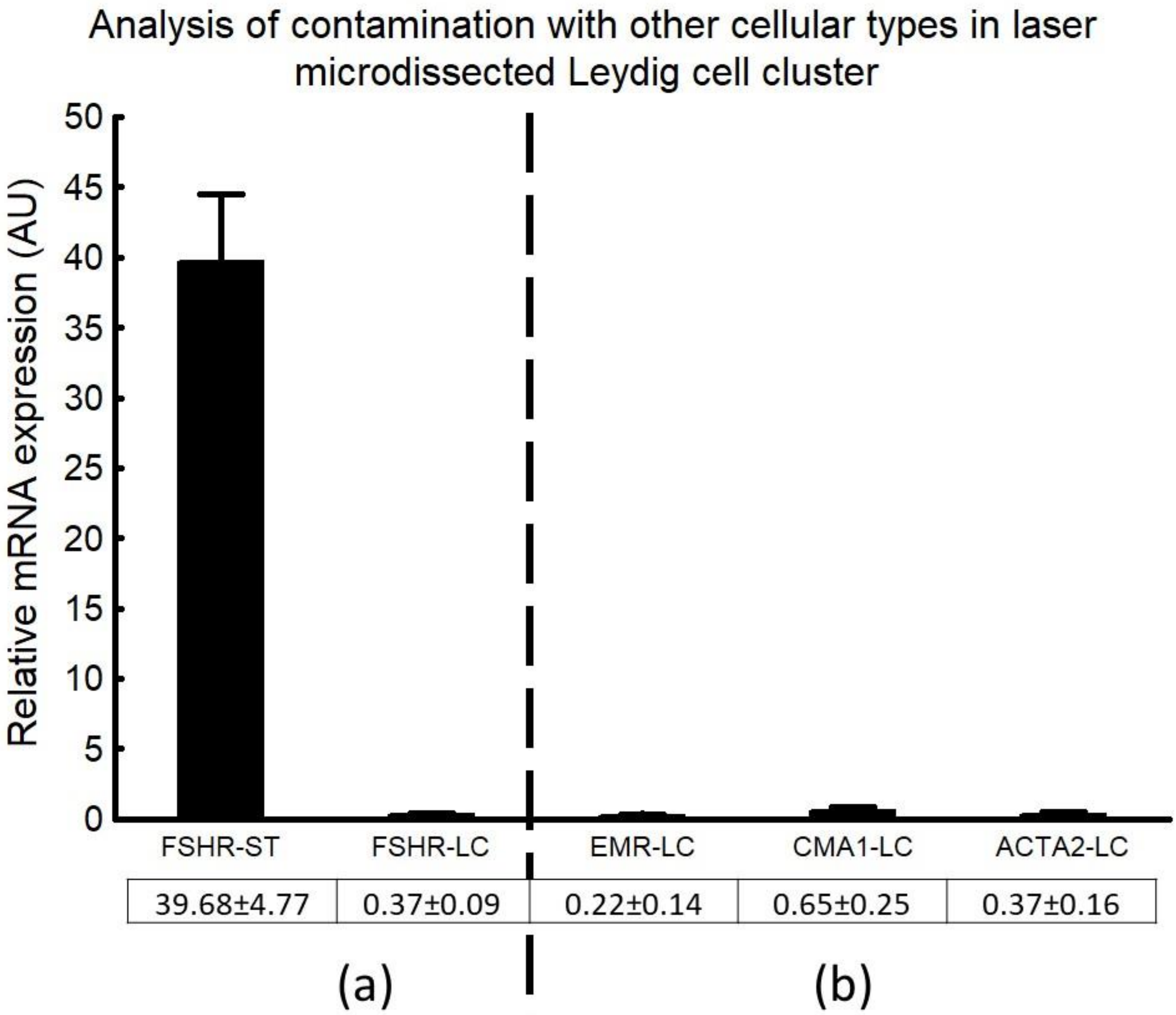

Figure S4. (a) Transcriptional levels of follicle stimulating hormone receptor (FSHR) in microdissected seminiferous tubules with complete spermatogenesis (FSHR-ST) and Leydig cell clusters (FSHR-LC); (b) transcriptional levels of EGF-like module receptor 1 (EMR1), chymase (CMA1) and α smooth muscle actin (ACTA2) in laser-microdissected Leydig cell clusters (LC). AU—arbitrary units (relative quantification normalized to the reference gene RPS29 using the ΔCt method). Data are presented as mean± SEM.
