# Supplementary material for: Expression of G-Protein-Coupled Estrogen Receptor (GPER) in Whole Testicular Tissue and Laser-Capture Microdissected Testicular Compartments of Men with Normal and Aberrant Spermatogenesis
Source: Biology (Basel). 2022 Feb 26;11(3):373. doi: 10.3390/biology11030373 (PMC8945034; doi:10.3390/biology11030373)
Supplement: Supplementary file 1 [file biology-11-00373-s001.zip › Figure S5.pdf]

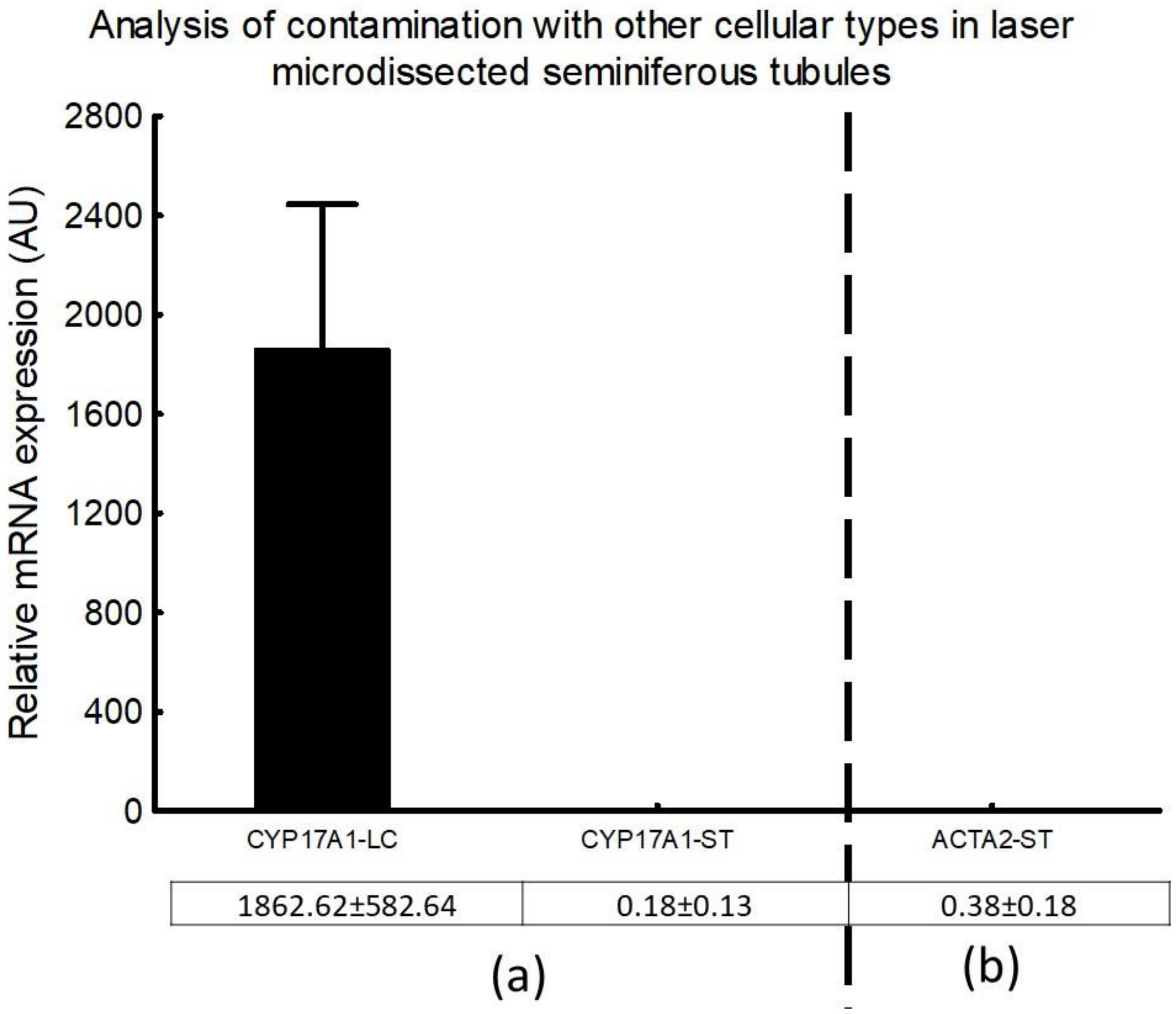

Figure S5. (a) Transcriptional levels of 17α-hydroxylase/17,20-lyase (CYP17A1) in laser-capture microdissected Leydig cell clusters (CYP17A1-LC) and seminiferous tubules with complete spermatogenesis (CYP17A1-ST); (b) transcriptional levels of α smooth muscle actin (ACTA2) in laser-microdissected seminiferous tubules (ST). AU—arbitrary units (relative quantification normalized to the reference gene RPS29 using the ΔCt method). Data are presented as mean± SEM.
