# Supplementary material for: Expression of G-Protein-Coupled Estrogen Receptor (GPER) in Whole Testicular Tissue and Laser-Capture Microdissected Testicular Compartments of Men with Normal and Aberrant Spermatogenesis
Source: Biology (Basel). 2022 Feb 26;11(3):373. doi: 10.3390/biology11030373 (PMC8945034; doi:10.3390/biology11030373)
Supplement: Supplementary file 1 [file biology-11-00373-s001.zip › Table S1.pdf]

Table S1. Clinical parameters and results of histological evaluation in the subgroups of men whose biopsies were subjected to seminiferous tubule (ST) laser microdissection.

	**OA-ST n=16**	**NOA-ST n=27**
Age (years)	31.0 (27.0-34.0)	32.0 (30.0-34.0)
Testicular volume (mL)*	15.0 (14.0-16.0)	9.5 (8.0-11.0)[a]
STD (μm)	200.0 (173.6-212.0)	141.5 (132.0-166.3)[a]
TM (μm)	5.8 (5.1-6.3)	7.9.0 (6.4-9.8)
LC-score (points)	1.8 (1.4-1.9)	2.4 (1.9-3.0)[a]

Values are median (interquartile range); Mann-Whitney U test, [a]$p<0.05$ with respect to OA-ST; LC – Leydig cells; n- number of subjects; NOA-ST – subgroup of men with non obstructive azoospermia and disturbed spermatogenesis, whose biopsies were subjected to seminiferous tubules laser microdissection, OA-ST – subgroup of men with obstructive azoospermia and complete spermatogenesis, whose biopsies were subjected to seminferous tubules laser microdissection; STD – seminiferous tubules diameter; TM- thickness of tubular membrane; *volume of the biopsied testis.
