# Supplementary material for: Expression of G-Protein-Coupled Estrogen Receptor (GPER) in Whole Testicular Tissue and Laser-Capture Microdissected Testicular Compartments of Men with Normal and Aberrant Spermatogenesis
Source: Biology (Basel). 2022 Feb 26;11(3):373. doi: 10.3390/biology11030373 (PMC8945034; doi:10.3390/biology11030373)
Supplement: Supplementary file 1 [file biology-11-00373-s001.zip › Table S2.pdf]

Table S2. Clinical parameters and results of histological evaluation in the subgroups of men whose biopsies were subjected to Leydig cell clusters (LC) laser microdissection

	**OA-LC n=11**	**NOA-LC n=14**
Age (years)	30.0 (26.0-33.0)	32.0 (30.0-34.0)
Testicular volume (mL)*	15.0 (12.0-16.0)	8.8 (8.0-11.0)[a]
STD (μm)	209.8 (180.0-217.6)	140.4 (126.5-160.0)[a]
TM (μm)	5.9 (5.1-6.3)	9.1 (7.7-10.4)
LC-score (points)	1.8 (1.3-1.9)	2.6 (2.1-3.0)[a]

Values are median (interquartile range); Mann-Whitney U test, $^{a}p<0.05$ with respect to OA-LM; LC – Leydig cells; n- number of subjects; NOA-LM – subgroup of men with non obstructive azoospermia and disturbed spermatogenesis, whose biopsies were subjected to Leydig cell cluster laser microdissection, OA-LM – subgroup of men with obstructive azoospermia and complete spermatogenesis, whose biopsies were subjected to Leydig cell cluster laser microdissection; STD – seminiferous tubules diameter; TM- thickness of tubular membrane; *volume of the biopsied testis.
