# Supplementary material for: Expression of G-Protein-Coupled Estrogen Receptor (GPER) in Whole Testicular Tissue and Laser-Capture Microdissected Testicular Compartments of Men with Normal and Aberrant Spermatogenesis
Source: Biology (Basel). 2022 Feb 26;11(3):373. doi: 10.3390/biology11030373 (PMC8945034; doi:10.3390/biology11030373)
Supplement: Supplementary file 1 [file biology-11-00373-s001.zip › Table S3.pdf]

Table S3. Serum hormonal profile in the subgroups of men whose biopsies were subjected to seminiferous tubule (ST) laser microdissection technique

	**OA-ST n=16**	**NOA-ST n=27**
FSH (mIU/mL)	3.3 (2.7-3.7)	14.9 (10.6-22.5)[a]
LH (mIU/mL)	3.8 (3.3-5.2)	6.8 (4.9-9.1)[a]
Testosterone (T) (nmol/L)	16.5 (9.4-19.7)	15.3 (11.6-18.4)
Estradiol (E) (pmol/L)	117.5 (55.7-157.5)	103.6 (80.2-124.1)
T/LH ratio	3.9 (2.7-5.5)	2.4 (1.8-3.1)[a]
E/T ratio	5.9 (4.8-8.0)	6.2 (4.9-8.3)

Values are median (interquartile range); Mann-Whitney U test, $^{a}p<0.05$ with respect to OA; n- number of subjects, NOA-ST – subgroup of men with non obstructive azoospermia and disturbed spermatogenesis, whose biopsies were subjected to seminiferous tubules laser microdissection, OA-ST – subgroup of men with obstructive azoospermia and complete spermatogenesis, whose biopsies were subjected to seminiferous tubules laser microdissection.
