# Supplementary material for: Expression of G-Protein-Coupled Estrogen Receptor (GPER) in Whole Testicular Tissue and Laser-Capture Microdissected Testicular Compartments of Men with Normal and Aberrant Spermatogenesis
Source: Biology (Basel). 2022 Feb 26;11(3):373. doi: 10.3390/biology11030373 (PMC8945034; doi:10.3390/biology11030373)
Supplement: Supplementary file 1 [file biology-11-00373-s001.zip › Table S4.pdf]

Table S4. Serum hormonal profile in the subgroups of men whose biopsies were subjected to Leydig cell cluster (LC) laser microdissection technique.

	**OA-LC n=13**	**NOA-LC n=14**
FSH (mIU/mL)	3.2 (2.4-3.7)	17.7 (14.0-22.5)[a]
LH (mIU/mL)	4.5 (2.5-5.8)	6.8 (5.7-8.1)[a]
Testosterone (T) (nmol/L)	14.0 (8.9-19.7)	16.1 (15.1-20.6)
Estradiol (E) (pmol/L)	113.2(42.8-157.5)	103.6 (82.2-164.5)
T/LH ratio	3.7 (2.7-5.5)	2.1 (1.8-2.9)[a]
E/T ratio	6.2 (4.8-8.0)	6.8 (4.8-7.9)

Values are median (interquartile range); Mann-Whitney U test, [a]$p<0.05$ with respect to OA-ST; n- number of subjects, NOA-ST – subgroup of men with non obstructive azoospermia and disturbed spermatogenesis, whose biopsies were subjected to seminiferous tubules laser microdissection, OA-ST – subgroup of men with obstructive azoospermia and complete spermatogenesis, whose biopsies were subjected to seminiferous tubules laser microdissection;
